# Impact of Hydrogel-to-Oleogel Ratio and Presence of Carob Fruit Extracts on Formulated Bigels: Rheological, Thermal, Physicochemical and Microstructural Properties

**DOI:** 10.3390/foods14213753

**Published:** 2025-10-31

**Authors:** María Dolores Álvarez, Arancha Saiz, Susana Cofrades

**Affiliations:** Institute of Food Science, Technology and Nutrition (ICTAN-CSIC), C/José Antonio Novais 6, 28040 Madrid, Spain; a.saiz@ictan.csic.es (A.S.); scofrades@ictan.csic.es (S.C.)

**Keywords:** bigel, sodium alginate, olive pomace oil, microcrystalline wax, carob extracts, inositols, phenolic compounds, rheology, thermal behavior, microstructure

## Abstract

This study explores the development of bigels (BGs) combining a hydrophilic hydrogel (HG) and a lipophilic oleogel (OG) for co-delivery of two carob fruit extracts (CFEs): I-CFE (inositols) and P-CFE (phenolics). The BGs were formulated in HG:OG ratios of 70:30 and 30:70, using a sodium alginate-based HG and an OG composed of olive pomace oil (OPO) and microcrystalline wax (MW). CFEs were loaded in three modes: I-CFE in HG, P-CFE in OG, and both in their respective phases. Rheological, thermal, physicochemical, and microstructural properties were assessed. All the BGs exhibited solid-like viscoelastic behavior, with greater rigidity in 30:70 formulations. The OG phase enhanced the structural BG network, especially when loaded with P-CFE. At 70:30, I-CFE conferred pseudoplasticity and conformational flexibility, particularly in the absence of P-CFE. At 30:70, both extracts acted synergistically, increasing mechanical strength and network organization. Thermal analysis confirmed MW’s role in structuration, with the BGs showing melting peaks between 40–50 °C. The effects studied affected color and stability. Polarized light microscopy confirmed organized microstructures. This is the first work demonstrating the structuring potential and interactive effects of dual carob extracts (I-CFE and P-CFE) within BGs. All the BGs showed suitable fat-replacer properties, remaining self-standing for 21 days, except the 70:30 I-CFE-free formulation. The findings highlight the potential of CFE-loaded BGs as multifunctional fat replacers in healthier meat products.

## 1. Introduction

The term *bigel* (BG) refers to a biphasic system composed of two structurally distinct gelled phases—an HG and an OG—which differ in polarity [1]. Both phases are characterized by three-dimensional networks formed by polymeric or crystalline gelators, resulting in limited fluidity. HGs and OGs are widely recognized as effective carriers for the encapsulation and controlled release of bioactive compounds. Therefore, the structural integrity of BGs is critical for the efficient entrapment and transport of hydrophilic and lipophilic molecules within the respective aqueous and lipid phases, either independently or simultaneously [2]. Although BGs were originally developed for pharmaceutical and cosmetic applications [2,3], their use has recently expanded to the development of innovative food products. This includes their incorporation as fat replacers [1], owing to their capacity to deliver functional ingredients—encompassing both polar and non-polar bioactive compounds—and to improve the stability of the final product [4,5,6,7,8], although their application remains at the laboratory scale.

The biphasic structure of a BG enables the delivery of a wide range of bioactive compounds (such as lipids, pigments, and vitamins). Additionally, the distinct physicochemical characteristics of each phase contribute to the controlled release of these substances while also offering enhanced protection against degradation. The encapsulation of various substances (e.g., vitamins, flavorings, and antioxidants) in BGs and the assessment of their controlled release and diffusion rates offer opportunities for the development of functional and sustainable new food products, meeting current and future consumer demands. Xie et al. [4] developed 3D-printed BGs using fish gelatin to form an HG and candelilla wax to produce an OG, aiming to deliver both hydrophilic quercetin and lipophilic catechin. Their study showed that both the HG:OG ratio and the type of emulsifier significantly influenced the extent of lipolysis and release behavior of the encapsulated compounds. Following in vitro digestion, the highest release rates were observed for catechin (53.37%) in BGs containing monoglyceride with 70% OG and for quercetin (11.08%) in BGs formulated with lecithin and 30% OG. Zheng et al. [5] formulated food-grade BGs as delivery systems for the lipophilic compound *β*-carotene. They observed that increasing the OG proportion to 75% significantly enhanced the release of *β*-carotene—reaching values between approximately 60% and 80%—during simulated intestinal digestion, which could contribute to improved absorption. Nevertheless, in novel BGs designed for lycopene delivery, which incorporated high-acyl gellan gum within the HG phase and a monoglyceride–beeswax blend within the OG phase [6], the authors concluded that a higher OG content could slow down the release of lycopene. In addition, BGs formulated with 25% OG—and therefore with a continuous HG phase—showed the greatest lutein release (83.2%) [7]. In turn, Loza-Rodríguez et al. [8] developed BGs aimed at stabilizing ascorbic acid against degradation caused by temperature and solar radiation.

The type of oil used as the solvent in the OG system also significantly influences the thermal, rheological, and mechanical properties, as well as the nutritional characteristics, of the resulting OGs and, consequently, the final properties of the BGs. Various vegetable oils—such as corn [5], soybean [9], sunflower [10], and olive [11]—have been incorporated into the OG phase of BGs. However, OPO has only been used in combination with sunflower oil in carnauba-wax-based OGs [12]. OPO is a refined oil obtained from alperujo, a by-product of virgin olive oil production. Spain, as the world’s leading producer of OPO, is making significant efforts to valorize this resource within a circular economy framework, promoting sustainability and zero-waste practices [13]. In addition, OPO stands out for its nutritional and functional profile, rich in oleic acid and minor bioactive compounds with health-promoting potential. Its composition makes it a promising alternative to conventional fats in food systems. Given its technological stability and sustainable origin, OPO is a suitable candidate for the lipid phase in BG formulations, contributing to the development of functional foods aligned with environmental and nutritional goals. For these reasons, OPO was selected in this study as the lipid phase of BGs, aiming to explore its potential as a sustainable and functional lipid component.

On the other hand, CFEs, which are rich in various bioactive derivatives, have been identified as promising dietary supplements due to their high fiber content and the presence of compounds with recognized antidiabetic properties [14]. Studies have shown that extracts obtained from carob pod by-products, enriched in bioactive constituents, may contribute to the prevention and management of type 2 diabetes mellitus [15]. The carob pod contains a diverse array of bioactive compounds, among which inositols (present in the I-CFE extract) and phenolic compounds (present in the P-CFE extract) are particularly notable for their antidiabetic potential. Indeed, the hypoglycemic effects of both I-CFE and P-CFE have been demonstrated in preclinical and clinical research [15,16]. These bioactive constituents exert their glucose-lowering effects through distinct mechanisms, suggesting a synergistic interaction that could be harnessed to enhance diabesity treatment strategies. All the properties attributed to I-CFE and P-CFE suggest that their combined administration could be of interest, as they may enhance each other’s efficacy. To our knowledge, this is the first study to incorporate both CFEs into a dual-phase BG system, enabling their co-delivery in a structured matrix. This biphasic approach not only may allow for the fine-tuning of release kinetics—specifically by slowing down the release of P-CFE compared to I-CFE during in vitro gastrointestinal digestion, thereby mitigating pharmacokinetic antagonism—but also enhance their applicability in meat products by improving physical stability and masking undesirable sensory attributes of P-CFE. However, no studies to date have described the differences in the effects of these two carob fruit extracts (I-CFE and P-CFE) when administered separately or in combination.

Bearing in mind that the properties of BGs are strongly influenced by technological parameters—such as the HG:OG ratio, the type and concentration of structural components in each phase, and the preparation and storage conditions [1]—prior knowledge and optimization of these variables are essential for designing BGs that incorporate an HG phase enriched with I-CFE and an OG phase containing P-CFE. These systems should also exhibit the functional properties required for their potential application as fat replacers in functional meat products. The main aim of this study was to characterize the mechanical and thermal properties, stability, and microstructure of BGs developed as dual delivery systems for CFEs, with the goal of subsequently enabling the controlled release of their bioactive constituents during digestion and evaluating their suitability as fat replacers.

## 2. Materials and Methods

### 2.1. Materials

I-CFE and P-CFE extracts were kindly provided by PlanTech BioTechnology Spain S.L. (Valencia, Spain) under the brand NOW^®^ (Nutrition Optimised Within). Sodium alginate (SA), containing 70% carbohydrates, was obtained from TRADES (Barcelona, Spain). MW was supplied by Iberceras (Madrid, Spain). Soy lecithin (Verolec Non GMO IP) was acquired from Lasenor Emul S.L. (Barcelona, Spain). Refined OPO was produced in Spain, donated by Interprofesional del Aceite de Orujo de Oliva (ORIVA, Sevilla, Spain), and refined by ACESUR S.A. (Sevilla, Spain).

### 2.2. Preparation of Bigels (BGs)

In this study, the BG preparation method was treated as a fixed factor, with all systems prepared by mixing the HG and OG phases prior to gel setting [3]. The main variables evaluated were the HG:OG ratio (100:0, 70:30, 30:70, 0:100) and the presence of CFEs—inositol-rich I-CFE and phenolic-rich P-CFE—in the individual phases. When present, I-CFE was incorporated into the HG phase (HG*), and P-CFE into the OG phase (OG*). Six BGs were formulated: three with a 70:30 HG:OG ratio (HG70*:OG30, HG70:OG30*, HG70*:OG30*) and three with a 30:70 ratio (HG30*:OG70, HG30:OG70*, HG30*:OG70*). The asterisk indicates that the corresponding phase was loaded with its respective extract (I-CFE for HG*, P-CFE for OG*), allowing the exploration of potential synergistic or antagonistic interactions. All BG formulations, along with the control phases (HG* and OG*), are summarized in Table 1. The 70:30 and 30:70 ratios were selected based on preliminary trials that also included 80:20, 20:80, and 50:50 proportions. The chosen ratios provided sufficiently distinct gel structures to enable differential release profiles of the bioactive compounds during gastrointestinal digestion.

At each HG:OG ratio, BGs were prepared by combining individually formulated phases: HG* (with I-CFE) with OG (without P-CFE), HG (without I-CFE) with OG* (with P-CFE), and both HG* and OG* (Table 1). To prepare 100 g of HG*, 98 g of I-CFE and 2 g of sodium alginate (SA) were mixed under stirring (400 rpm, 30 min). For HG, 98 g of distilled water replaced the I-CFE, maintaining the same SA content and conditions. OG* was prepared by heating 85.25 g of OPO, 12.5 g of MW, and 2 g of P-CFE at 70 °C until fully dissolved, followed by the addition of 0.25 g of soy lecithin and homogenization with an Ultra-Turrax at 9000 rpm for 1 min. OG was prepared similarly, omitting the extract and adjusting OPO to 87.25 g. Extract concentrations were standardized based on specific bioactive content (inositols and phenolic compounds), although no official recommendations exist for optimal dosing in structured food systems.

The required amounts of each phase were weighed according to the target ratio (70:30 or 30:70), with all BG batches standardized to 100 g. Specifically, 70 g of HG and 30 g of OG were used for the 70:30 ratio, and vice versa for the 30:70 ratio. To form the BG, OG was first homogenized (Ultra-Turrax, 9000 rpm, 30 s), followed by the addition of HG, and both phases were homogenized together (9000 rpm, up to 4 min). The final BGs were transferred into sterile containers and stored at 4 °C for 72 h prior to use to allow proper gel setting. A detailed description of the exact ingredient quantities for each phase and BG formulation, according to ratio and extract presence, is provided in Table 2.

### 2.3. Rheological Characterization

All rheological measurements were done using a stress-controlled Rheometer Kinexus pro (NETZSCH-Gerätebau GmbH, Wittelsbacherstr, Selb, Germany) at 25 °C. Temperature was controlled by using a controller cartridge holding the lower plate on which the sample is placed. A cover was used to maintain the samples at specified temperature. Sandblasted parallel plate-plate geometry of 20 mm diameter and a gap of 1.5 mm was used for the individual OG* phase and the BGs, whereas a 40 mm cone-plate geometry (2° angle and target gap of 0.0700 mm) was used for the individual HG* phase. Samples were always evaluated in triplicate.

#### 2.3.1. Oscillatory Measurements

Before measurements at 25 °C, all systems were allowed to rest on the geometry for 25 min (to stabilize and restructure the sample) using time sweeps carried out at 1 Hz with a previously chosen stress within the linear viscoelastic region (LVR). Then, in order to determine the extent of the LVR, amplitude stress sweeps were carried out at 1 Hz varying the stress depending on the material measured, typically from 0.05 to 5 Pa for BGs at 70:30 ratio, and from 1 to 100 Pa for BGs at 30:70 ratio. Then, frequency sweeps were performed between 10 and 0.1 Hz at chosen stress within the LVR to ensure that the material response in terms of storage or elastic modulus (*G*′, kPa) and loss or viscous modulus (*G*″, kPa) was independent of the strain magnitude. Furthermore, complex modulus (G* = [(*G*ʹ)^2^ + (*G*″)^2^]^0.5^), from frequency sweeps, was fitted to the power law known as the weak gel model [17] (Equation (1)):

(1) $$G^{*}\left(f\right)=Af^{1/z}$$

where G* is the complex modulus in kPa, *f* is the frequency in Hz, *A* (kPa s^1/*z*^) is the proportional coefficient or strength of the interactions (G* at 1 Hz), and *z* (dimensionless) is the coordination number or network extension; it can be regarded as a measure of structural degree [18].

#### 2.3.2. Steady-State Measurements

Samples were placed on the corresponding geometry and allowed to rest for 5 min before analysis at 25 °C to ensure both thermal and mechanical equilibrium. Then, a pre-shearing test was conducted on each sample over 1 min at 100 s^−1^, and immediately a flow curve was obtained as a function of shear rate ranging between 100 to 0.01 s^−1^ with 5 points per decade for either BGs or for the individual HG* and OG* phases. The power law (Equation (2)) was used to describe the effect of the shear rate on apparent viscosity for the shear stress data pertaining to the samples:

(2) $$\eta \left(\dot{\gamma }\right)=K{\dot{\gamma }}^{n-1}$$

where *η* is the apparent viscosity (Pa s), $\dot{\gamma }$ is the shear rate (s^−1^), *K* is the consistency index (Pa s^n^), and it is equivalent to apparent viscosity at 1 s^−1^, and *n* is the flow behavior index (dimensionless) reflecting the closeness to Newtonian flow. Apparent viscosities (Pa s) in fixed shear rates of 10 s^−1^, indicator of oral shear rate [19], 50 s^−1^ (referenced by the National Dysphagia Diet [20]), and of 0.1 and 100 s^−1^, as representative values of low and high shearing processes, were evaluated for the BGs and both individual HG* and OG* phases.

### 2.4. Thermal Behavior

A TA Q1000 differential scanning calorimeter (TA Instruments, New Castle, DE, USA) was used to evaluate the thermal characteristics of the different systems. A 15–20 mg amount of each sample was placed in an aluminum pan and hermetically sealed. An empty pan was used as a reference. For heating thermograms, the samples were heated from 0 to 80 °C with a constant heating rate of 5 °C/min. The onset and peak temperatures (*T*_O_ and *T*_P_, °C) and the enthalpy of the melting point (Δ*H*_M_, J/g) were recorded for the BGs and the individual OG* phase, as well as for OPO and MW, using Universal Analysis 2000. Samples were always evaluated in triplicate.

### 2.5. Color Measurement, Physical Stability and Total Loss

Color analysis of the BGs was performed using a Konica Minolta CM-3500D spectrophotometer (Konica Minolta Business Technologies, Tokyo, Japan), placed on a glass plate and configured with a D65 illuminant and a 10° standard observer. The parameters recorded included lightness (L*), redness (+a*), yellowness (+b*), and blueness (−b*) to determine the color coordinates. Each sample was measured ten times.

The tube inversion test was used to evaluate the physical stability of the BGs after 72 h and three weeks of storage at 4 °C following their preparation. For this purpose, approximately 10 g of BG samples were placed in 50-mL Falcon tubes, and, at 3 and 21 days post-preparation, the tubes were kept in an inverted position for 1 h to confirm their gel-like behavior.

To evaluate total loss, approximately 2 g of BG sample were placed in Eppendorf tubes and stored under refrigeration at 4 °C for 72 h. After this period, the samples were centrifuged at 10,000 rpm for 15 min at room temperature using a Heraeus Multifuge 3 L-R centrifuge (Kendro Laboratory Products, Osterode am Harz, Germany). The tubes were then opened and placed in an inverted position over aluminum capsules for 30 min to collect the exudate (including water and fat). The total loss (%)—comprising the combined release of fat and water—was determined by calculating the difference between the initial and final weight of the Eppendorf tube (after 30 min), divided by the BG sample weight. This measurement was performed at least in triplicate.

### 2.6. Microscopy

The microstructure of the BGs was visualized using polarized light microscopy (PLM) on a Leica AF6000 LX system microscope (Mannheim, Germany), housed in a Pecon environmental chamber maintained at 37 °C (Promi III Pol, Carl Zeiss AG, Germany). Images were captured with a Hamamatsu C9100-02 digital camera. Approximately, 15 mg of each refrigerated BG sample was placed at the center of a glass slide and covered with a coverslip. The slides were then stored at 4 °C for 72 h to allow cooling and recrystallization before microstructural analysis. Imaging was performed using 10×/0.30 NA and 40×/0.75 objective lenses, with an additional 1.6× magnification and illumination provided by an Hg-arc lamp. Images were saved at a resolution of 1000 × 1000 pixels using the Leica Application Suite X (LAS X) software, version 5.3.1 (Leica, Microsystems, Johnson City, TN, USA).

### 2.7. Statistical Analysis

All measurements mentioned above were repeated at least in triplicate, using consistent batch preparations, and therefore refer to technical replicates. The results were expressed as mean ± standard deviation. One-way analysis of variance (ANOVA) was used to highlight the effect of I-CFE and P-CFE extracts on the rheological, thermal and physicochemical properties of BGs at the same 70:30 or 30:70 HG:OG ratio. Due to the substantial differences observed in the rheological properties of BGs at both 70:30 and 30:70 ratios, statistical comparisons of the different properties evaluated were not conducted between them in order to avoid masking the effect of the presence or absence of the CFEs within the characteristics of the BG systems. Values of different properties for HG* and OG*, as well as for I-CFE and P-CFE are provided as references; however, they were not included in the statistical analysis. Significant differences between pairs of means were evaluated by the Tukey test, using a 95% confidence interval (*p* < 0.05). Analyses were performed using IBM SPSS for Windows, Version 27.0 (IBM Corp., Armonk, NY, USA).

## 3. Results and Discussion

### 3.1. Effects of HG:OG Ratio and I-CFE and P-CFE Extracts on Rheological Properties of Bigels

#### 3.1.1. Oscillatory Measurements

**Figure 1:**
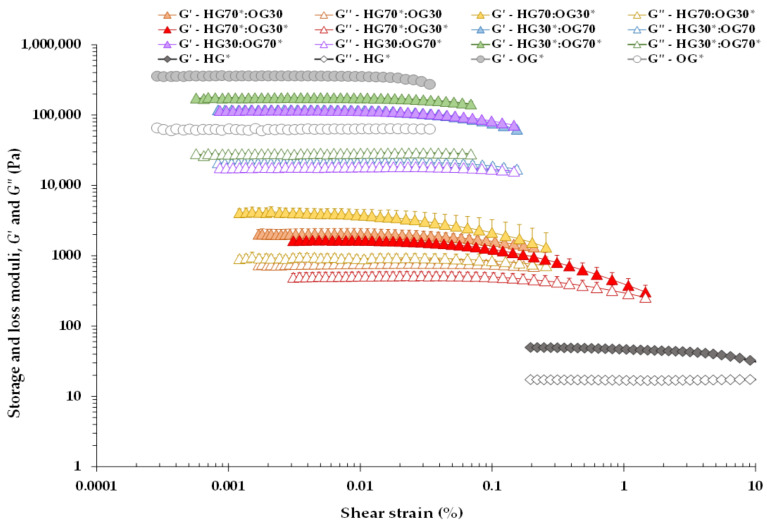
Storage modulus (*G*′) and loss modulus (*G*″) at 25 °C for bigels prepared at 70:30 and 30:70 ratios, as well as for the individual hydrogel (HG*) and oleogel (OG*) phases, as a function of the applied shear strain at 1 Hz.

Figure 1 shows the storage (*G*′) and loss (*G*″) moduli of the different BGs, as well as those of the individual HG* and OG* phases. HG* incorporates I-CFE, while OG* incorporates P-CFE. All systems exhibited a plateau within the linear viscoelastic region (LVR), where *G*′ consistently exceeded *G*″, indicating structured networks. OG* showed the highest moduli, HG* the lowest, and BGs intermediate values. BGs with a 30:70 ratio exhibited significantly higher moduli than those with a 70:30 ratio.

OG* and 30:70 BGs had a lower LVR (strain < 0.03%) compared to HG* and 70:30 BGs, which reached strain values above 1.00% (Figure 1). These results align with previous studies [7], indicating OG* has a brittle microstructure, while HG* is creamier. BGs exhibited intermediate behavior modulated by the nature of the discrete phase. BGs with 70% HG adopted an OG-like dispersed phase within a continuous HG matrix, showing higher strain tolerance. Conversely, BGs with 70% OG had a dispersed HG phase and lower LVR. In both ratios, BGs resembled OG* more closely, suggesting a dominant influence of the OG phase.

Table 3 presents rheological parameters defining the LVR limits. *σ*_max_ indicates structural stability; *γ*_max_ relates to conformational flexibility; G*_max_ reflects network rigidity; and tan *δ* (*G*″/*G*′) indicates viscoelasticity [21,22,23]. HG showed *γ*_max_ of 2.80%, OG* only 0.03%, and BGs ranged from 0.01–0.10% (70:30) and 0.02–0.03% (30:70). BGs with higher OG content had conformational flexibility similar to OG*, while 70:30 BGs showed increased flexibility when HG was loaded with I-CFE. At this ratio, I-CFE accounted for 68.60% of BG composition (Table 2), contributing to a creamy consistency.

These results are consistent with previous studies [7], where ethyl cellulose-based OG exhibited a smaller LVR (0.01–0.04%) than HG formulated with a guar–xanthan gum mixture (0.01–14.07%), both loaded with lutein. More recently, Richterová et al. [24] reported strain values at the end of the LVR ranging from 0.75% to 5.11%, depending on concentration, for agarose–collagen HGs. Overall, a decrease in the LVR and an increase in *G*′ and *G*″ were observed with increasing OG concentration (Figure 1). Additionally, compared to the individual loaded HG*, in all BGs—both when the OG was the dispersed phase (70:30 ratio) and especially when it was the continuous phase (30:70 ratio)—the OG, whether loaded or not, acted as an “interacting filler” [25], enhancing the rheological properties of the HG and promoting greater system structuration (e.g., significantly higher G*_max_ values; Table 3).

Similarly, Loza-Rodríguez et al. [8] reported critical strain values of 0.10% for BG and 3.2% for HG, although OG was not evaluated separately. Their BG (40:60 ratio) combined a lipid–water HG with an OG of olive oil and 6% beeswax, concluding that beeswax strongly influenced BG rheology. In our study, HG* is a soft sodium alginate (SA) gel with I-CFE, and BG behavior was mainly affected by MW in the OG phase—3.75% at 70:30 and 8.75% at 30:70 (Table 2)—regardless of P-CFE. OG* showed the highest *σ*_max_ and G*_max_ and the lowest tan *δ* (Table 3), confirming wax’s key role [8]. BGs at 30:70, with more OG and MW, exhibited significantly greater mechanical resistance and viscoelasticity than those at 70:30. Similar trends were observed in BGs with ethyl cellulose for lutein delivery [7] and in fat-replacer BGs for model meat products [10]. Additionally, increasing OG concentration (40–90%) led to a matrix-in-matrix structure, with flaxseed–beeswax OG and pectin HG phases entrapped within each other [26], reinforcing BG structuration. In contrast, BGs with beeswax-based OG and sodium alginate HG showed reduced textural properties at higher OG:HG ratios, suggesting that OG disrupted the alginate network [27].

In BGs with a 70:30 ratio, the presence of only the P-CFE extract in the OG significantly increased the overall resistance to elastic deformation and viscoelasticity (lower tan δ value) of the HG70:OG30* BG (Table 3), compared to BGs containing only the I-CFE extract or both extracts. In contrast, the conformational flexibility and stability of this BG were lower. It is likely that, at the 70:30 ratio, the amount of I-CFE present (68.60%) was excessive compared to the amount of P-CFE (0.60%) (Table 2), which may have hindered the development of a synergistic effect between the two extracts. Conversely, at the 30:70 ratio, the presence of both I-CFE and P-CFE extracts (at 29.40% and 1.40%, respectively) significantly increased the G*_max_ value of the HG30:OG70 BG compared to BGs containing only one extract (Table 3), indicating that at this ratio, with a higher OG fraction, both extracts appear to exert a synergistic effect that contributes to structuration.

**Figure 2:**
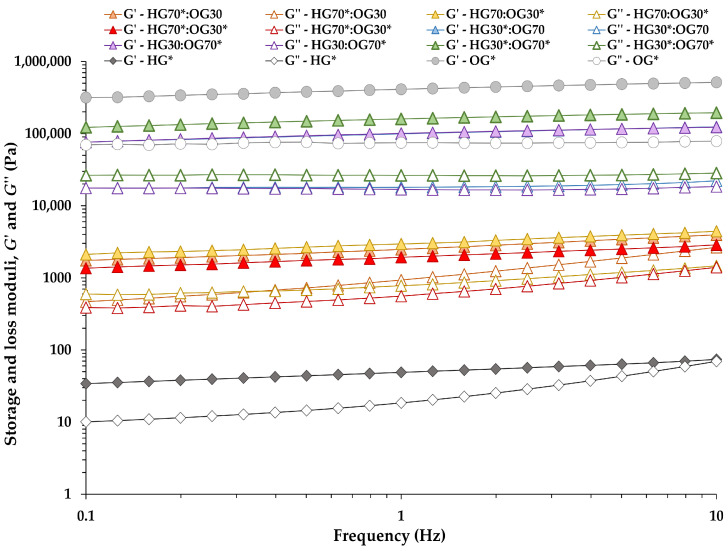
Storage modulus (*G*′) and loss modulus (*G*″) at 25 °C for bigels prepared at 70:30 and 30:70 ratios, as well as for the individual hydrogel (HG*) and oleogel (OG*) phases, as a function of the applied frequency at a selected stress within the LVR.

Frequency sweep tests (Figure 2) revealed a weak frequency dependence for OG* and all BGs, as both the *G*′ and *G*″ curves showed only slight positive slopes, even at higher oscillation frequencies. Additionally, the *G*′ values consistently exceeded the *G*″ ones across the entire frequency range for all these samples. The absence of crossover points between *G*′ and *G*″ further supports their predominantly elastic, solid-like behavior and suggests the formation of a gel network structure [5]. However, at the 70:30 ratio, the pronounced frequency dependence of *G*″ observed in the three BGs (Figure 2) may reflect the behavior of a fluid with yield stress [28,29], likely due to the dispersion of the oil phase as small droplets within the continuous HG matrix. Although yield stress was not directly measured in this study, it is plausible that both yield stress and gel stiffness increased with higher oil phase content at the 30:70 ratio. This enhancement is attributed to the strengthening of gel network cross-links in the BGs, as also supported by PLM images. In contrast, the HG* sample exhibited a more pronounced frequency-dependent behavior, with *G*″ values tending to approach *G*′ values at higher frequencies (Figure 2). It is worth noting that HGs lacking I-CFE extract exhibited rheological behavior more characteristic of polymer solutions, with *G*″ values exceeding *G*′ across the frequency range. However, upon formation of the BG system, this behavior was masked by the presence of the OG phase, resulting in a gel-like structure with mechanical strength comparable to that of BGs loaded with I-CFE extract at both 70:30 and 30:70 ratios. Interestingly, similar findings were reported by Kavya et al. [26], who developed BGs using a pectin-based HG. The authors showed that pure HG predominantly exhibited viscous behavior throughout the 0.1–100 Hz frequency range investigated, whereas pure OG and BGs displayed elastic behavior. These results confirm that the formulated BGs possess greater gel strength than the individual HG*, supporting their potential application as fat replacers.

As shown in Figure 2, both *G*′ and *G*″ values increased notably with higher OG content in the BGs, reflecting a greater degree of rigidity and structural organization. As previously mentioned, this can be attributed to the higher MW content (8.75%) and the increased volume of OPO droplets (Table 2). Consistent with our findings, improvements in viscoelastic properties and a dominant elastic response with increasing OG content have been reported in BGs formulated with ethyl cellulose (as oleogelator) and wheat starch (as hydrogelator) [10]. Similar trends were also observed in BGs prepared with varying HG:OG ratios using κ-carrageenan-based HGs and monoglyceride-based OGs [5]. In contrast, BGs composed of gelatin-based HGs and OGs made from beeswax and rice bran wax at different ratios [18] exhibited rheological behavior primarily governed by the HG phase. These observations suggest that the structural contribution of each individual phase is strongly influenced by the specific biopolymer and lipid components employed.

The rheological properties and parameters obtained from the frequency sweep tests are presented in Table 4. In BGs with a 70:30 ratio, although the OG was the dispersed phase, the HG70:OG30* BG formulated with OG* loaded with P-CFE exhibited a significantly higher elastic response (i.e., higher *G*′ and lower tan *δ* values) than the other two BGs at the same ratio (Table 4), which were developed using HG* loaded with I-CFE. Similarly, in BGs with a 30:70 ratio, the presence of both extracts in their respective individual phases (HG30*:OG70* BG) significantly increased the rheological moduli values. OG* also showed the highest *G*′ and *G*″ values among all samples, confirming its strong contribution to the overall structuration and mechanical strength of the BGs.

**Figure 3:**
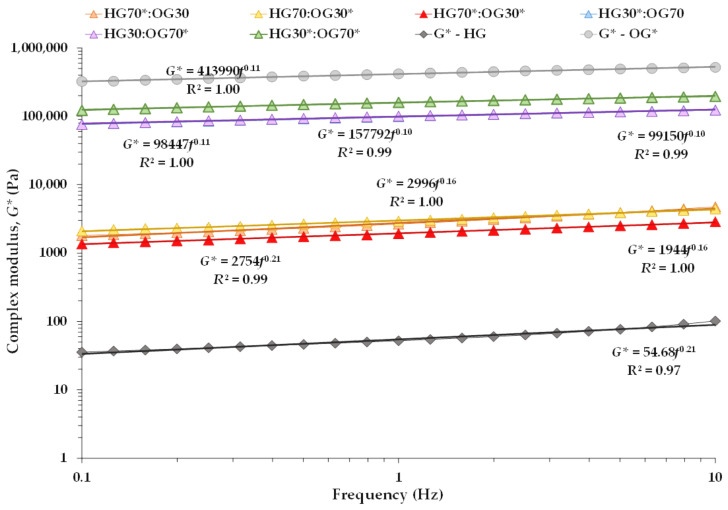
Complex modulus (G*) at 25 °C for bigels prepared at 70:30 and 30:70 ratios, as well as for the individual hydrogel (HG*) and oleogel (OG*) phases, as a function of the applied frequency at a selected stress within the LVR, along with potential fits to the weak gel model.

The mechanical spectra were also analyzed using the weak gel model [13,17,18]. Figure 3 shows the G* data of the BGs and the individual systems fitted to the weak gel model (Equation (1)), from which the parameters *A* and *z* were derived (Table 4). The gel strength (*A* value) of HG* was lower than that of the BGs (Table 4). Among the BGs with a 70:30 ratio, the HG70:OG30* sample showed the highest *A* value, confirming that the presence of P-CFE in the OG reinforced the three-dimensional matrix formed with a higher HG content. This suggests that P-CFE in the OG strengthened the polymeric structure through physical interactions with the HG matrix. From a mechanical perspective, such interactions likely increased the cross-linking density and network connectivity, resulting in a higher *A* value and more pronounced elastic behavior, as also evidenced by the higher *G*′ and lower tan *δ* values (Table 4). These interactions are presumed to be non-covalent in nature, including hydrogen bonding between phenolic hydroxyl groups and alginate chains, hydrophobic interactions between lipophilic domains, and Van der Waals forces that stabilize the interface between HG and OG phases. This compensatory effect was particularly relevant in the HG70:OG30* formulation, whose HG phase lacked I-CFE extract.

Also, the *A* value of pure OG* was noticeably higher than those of all BGs, which may be attributed to an optimized crystal structure in the absence of the HG phase—a more regular and compact arrangement that significantly enhances mechanical strength. However, the coordination number (*z*) values for BGs at 30:70 containing OG* were slightly higher than that of OG*, indicating greater internal structural stability in the presence of the HG, regardless of whether it was loaded with I-CFE. This suggests that while P-CFE reinforces the composite through external interactions, its presence in the absence of the HG phase may slightly disrupt the intrinsic crystalline packing of the OG, resulting in a lower *z* value.

As expected, both *A* and *z* values were also higher for BGs formulated at the 30:70 ratio than at 70:30 (Table 4), indicating a stronger and more interconnected gel network when the OG phase predominates, confirming previous findings [26]. Among the BGs, HG70:OG30* and HG30*:OG70* exhibited the highest *A* values at the 70:30 and 30:70 ratios, respectively. However, the *z* values of these two systems were similar to those of HG70*:OG30* and HG30:OG70*, suggesting that while gel strength was enhanced by the presence of the P-CFE extract, the I-CFE extract did not significantly affect the internal network connectivity. HG* with I-CFE had low gel strength but similar *z* value to BGs at 70:30 ratio, suggesting that 30% OG may not be enough to improve network stability. The weak gel formation is likely due to limited interactions between I-CFE and sodium alginate.

#### 3.1.2. Steady Shear Viscosities

**Figure 4:**
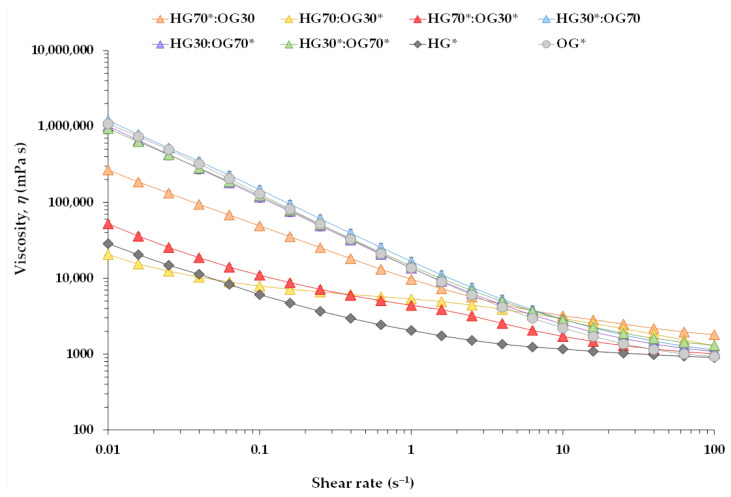
Viscosity (*η*) at 25 °C for bigels prepared at 70:30 and 30:70 ratios, as well as for the individual hydrogel (HG*) and oleogel (OG*) phases, as a function of the applied shear rate.

Figure 4 displays the apparent viscosity of the BGs, along with that of the HG* and OG* phases, under varying shear rates. In all cases, as the shear rate increased, the viscosity of the systems decreased, indicating a non-Newtonian, shear-thinning behavior. This was further supported by the flow behavior index (*n* < 1) obtained from the power law model, which ranged from 0.20 to 0.74 for the BGs (Table 5). Such behavior reflects the structural rearrangement of molecular chains under shear stress, which reduces internal friction and enhances fluidity.

However, noticeable differences were observed in the viscosities of the BGs at different ratios, as well as between the BGs and the individual OG* and HG* phases, particularly at lower shear rates. As shown in Figure 4, OG* exhibited a much higher viscosity than HG* at lower shear rates. However, from 10 s^−1^ onward, the viscosities of pure HG* and OG* tended to converge, displaying more similar values across the range of higher shear rates. A similar trend was observed in most of the homologous 70:30 and 30:70 BGs—i.e., those containing the same extract combinations.

Table 5 presents the apparent viscosity values at various shear rates, along with the consistency index (*K*) and the flow behavior index (*n*) for each system. For example, the viscosity at 0.1 s^−1^ of the HG30*:OG70 formulation—comprising HG* with I-CFE extract and OG without P-CFE—was higher than that of HG70*:OG30. Moreover, increasing the OG* fraction led to an increase in the *K* value corresponding to the shear rate of 1 s^−1^ (Table 5). These results suggest that OG* and BGs at the 30:70 ratio form a more rigid crystalline network compared to BGs at the 70:30 ratio and to HG* alone. However, the highest viscosities at 10 s^−1^ (a shear rate representative of oral processing) and at 50 s^−1^ (commonly used as a reference for dysphagia-oriented products) [19], as well as at 100 s^−1^, were observed in the HG70*:OG30 BG. This suggests that in the presence of a higher proportion of MW, the network structure collapses more rapidly under high shear stress. These findings are consistent with previous observations reported by Jiang et al. [30]. Interestingly, in both HG:OG ratios, the highest *K* values and the lowest *n* values—indicating more pronounced pseudoplastic behavior—were observed when the HG contained the I-CFE extract and the OG lacked the P-CFE extract (Table 5). This suggests that the presence of I-CFE in the HG* phase may contribute to a more uniform distribution of OPO droplets in the absence of P-CFE, thereby enhancing the consistency and structural integrity of the BGs at both ratios—even more so than when both extracts are present.

This result may appear to contrast with the oscillatory rheological results, where BGs containing P-CFE exhibited higher gel strength and viscoelastic moduli. However, oscillatory tests, performed within the LVR, assess the material’s elastic and structural behavior under small deformations, providing insights into the internal network connectivity and gel strength. In contrast, steady shear measurements reflect the flow behavior under continuous deformation, which is more sensitive to phase dispersion, droplet distribution, and overall homogeneity. Therefore, while P-CFE may enhance the gel network through physical interactions with the polymer matrix, I-CFE appears to improve the flow consistency and pseudoplasticity of the system, particularly when P-CFE is absent.

### 3.2. Effects of HG:OG Ratio and I-CFE and P-CFE Extracts on Thermal Properties of Bigels

**Figure 5:**
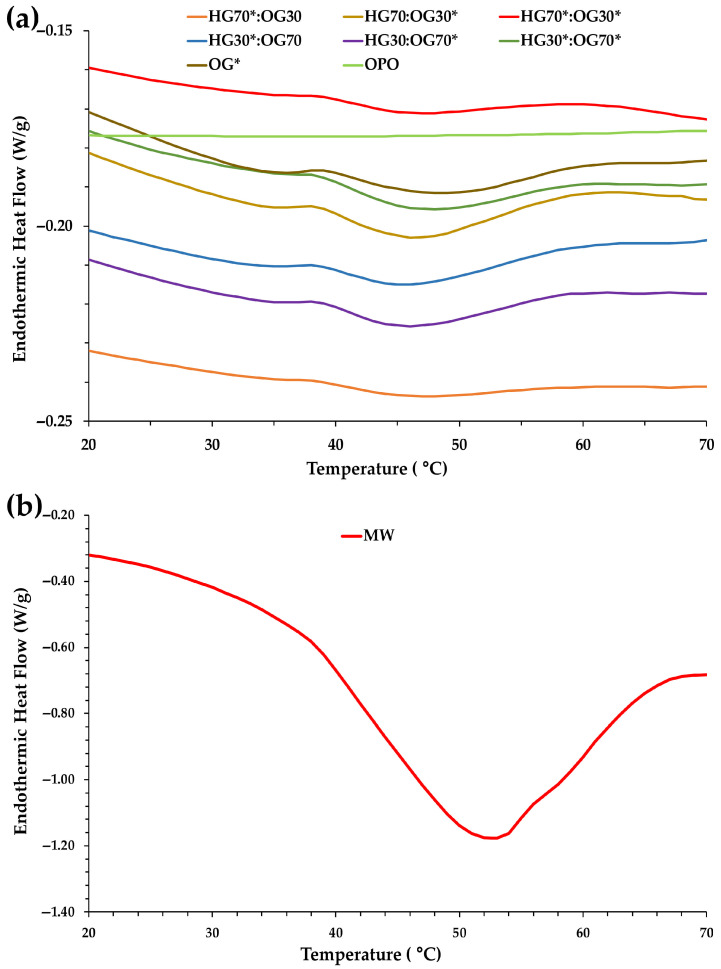
Melting profiles obtained by DSC: (**a**) For bigels (BGs) formulated at 70:30 and 30:70 ratios, individual oleogel (OG*) phase, and olive pomace oil (OPO); (**b**) For microcrystalline wax (MW).

Figure 5 presents the DSC heating thermograms of the BGs formulated at 70:30 and 30:70 ratios, incorporating the various combinations of I-CFE and P-CFE extracts. It also includes the melting profiles of OG*, OPO, and MW. All BGs exhibited a distinct endothermic peak between 40 and 50 °C (Figure 5a), which was slightly more pronounced in the 30:70 ratio. This peak is clearly attributed to the melting of MW incorporated into the OGs. In contrast, pure MW displayed a sharper and broader peak between 40 and 65 °C (Figure 5b). The OG* sample showed an intermediate endothermic peak between 40 and 60 °C (Figure 5a), positioned between those of the BGs and MW. Taken together, the results suggest a correlation between the thermal properties and the structural modifications occurring within the BG systems.

The melting of MW involves a phase transition from solid to liquid, accompanied by significant heat absorption, resulting in a prominent endothermic peak in the thermogram (Figure 5b). The OG* formulation contained 12.5 g of MW and 85.25 g of OPO, leading to a dilution effect that reduced the intensity of the absorption peak compared to pure MW. In the BGs, MW accounted for 3.75% and 8.75% of the total composition in the 70:30 and 30:70 ratios, respectively (Table 2). This lower wax content explains the smoother melting behavior observed in the BGs, particularly in the 70:30 formulation (Figure 5a), where the reduced amount of MW results in a diminished melting peak relative to OG* and pure MW.

Notably, no thermal event was detected in the heating curve of OPO (Figure 5a), confirming the high thermal stability of this olive oil category, as previously reported by Álvarez et al. [13].

The melting onset and peak temperatures, along with the enthalpies derived from the thermograms, are presented in Table 6. For the same HG:OG ratio, no significant differences were observed in the onset and peak temperatures, which were also very similar among the six BG formulations. This indicates that the different proportions of MW present at each ratio (3.75% and 8.75% for the 70:30 and 30:70 ratios, respectively) had no effect on the absorption peaks of the BGs. Pure MW exhibited the highest endothermic peak temperature (51.42 °C), while a slight leftward shift was noted in the endothermic peak of OG* (50.88 °C).

Regarding the enthalpy values, although they were much lower in all BGs compared to that of MW, at the 70:30 ratio this thermal parameter was significantly higher in the BG that did not contain the I-CFE extract in the HG phase (HG70:OG30*; Table 6). This suggests that when I-CFE represents 68.60% of the BG composition, it alters molecular interactions and the structural organization within the gel matrix, resulting in a less compact and less total energetically stable crystalline arrangement—i.e., lower lattice energy. In other words, the cohesive energy for the packing of molecules in the crystal structure of MW is larger in I-CFE absence at 70:30 ratio.

In contrast, at the 30:70 ratio, where I-CFE accounts for only 29.40% of the BG composition (Table 2), a synergistic effect between both carob extracts appears to emerge, as reflected by the higher enthalpy observed in HG30*:OG70* (Table 6). These thermal results are consistent with the enhanced rheological properties observed for these two BGs, suggesting that both the extract composition and the HG:OG ratio play a key role in modulating the structural integrity and energy profile of the bigel systems.

In the preparation of BGs, wax is commonly employed to structure OGs [6,8,18,26,27]. However, OGs formulated with wax typically exhibit high melting points, which can limit their applicability and performance in practical settings. For instance, DSC curves of BGs containing beeswax and rice bran wax have shown endothermic peaks at approximately 63 °C and 80 °C, respectively [18]. In turn, rice bran wax, carnauba wax, and candelilla wax had *T*_p_ values at 76, 84 and 64 °C, respectively [31]. Therefore, the BGs developed in the present study, incorporating MW characterized by a lower melting point, can address this limitation. The lower melting point of MW-based BGs (around 50 °C) is expected to facilitate better integration into meat matrices during cooking and processing, improve mouthfeel, and enhance the release of bioactive compounds during digestion—making them a promising fat replacer in meat products. While waxes like carnauba or beeswax are esters of a fatty alcohol and fatty acid, MWs are composed primarily of hydrocarbons, which could justify this finding.

### 3.3. Effects of HG:OG Ratio and I-CFE and P-CFE Extracts on Physicochemical Parameters of Bigels

**Figure 6:**
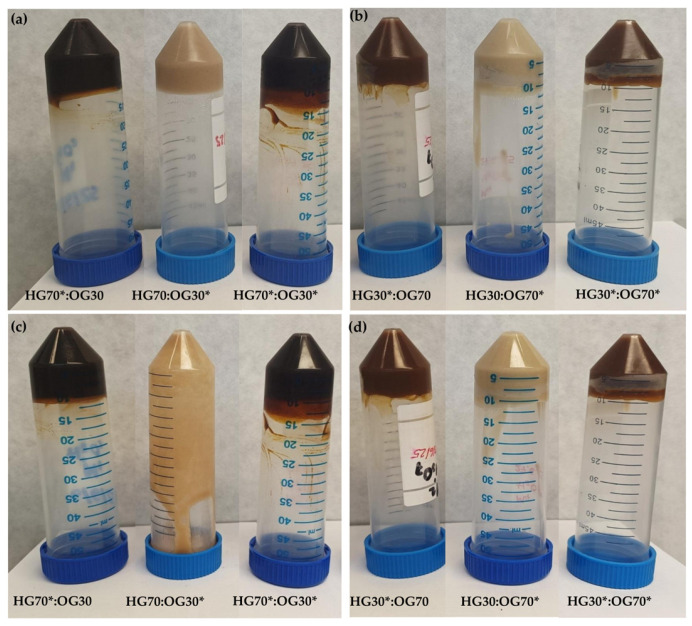
Visual appearance of bigels (BGs): (**a**) Inverted vial test images of BGs formulated with a 70:30 HG:OG ratio on day 3; (**b**) BGs formulated with a 30:70 HG:OG ratio on day 3; (**c**) BGs formulated with a 70:30 HG:OG ratio on day 21; (**d**) BGs formulated with a 30:70 HG:OG ratio on day 21.

Results indicated a change in the color of the BGs due to both the effect of the HG:OG ratio and the presence or absence of I-CFE and P-CFE extracts. This is mainly due to that the I-CFE extract, incorporated into the HG*, is a dark brown, viscous liquid, whereas the P-CFE extract, incorporated into the OG*, is a fine, brownish-yellow powder. The L*, a*, and b* values for each extract, as well as for the six BGs, are shown in Table 7. As a consequence of the aforementioned color of the extracts themselves, I-CFE has lower lightness (L* value), redness (a* value), and yellowness (b* value) than P-CFE. The inverted vial test images of the BGs also allow us to appreciate the color differences among the various BG formulations (Figure 6). It should be noted that, despite the presence of other ingredients in the individual HG and OG phases, the color parameters of all BGs remained practically within the range of those corresponding to the aforementioned extracts, once again highlighting their significant influence on the color of these systems. Specifically, SA is a fine white powder, MW exhibits a whitish-gray hue, soy lecithin is a viscous brown liquid, and OPO presents a light-yellow color. These components are consistently present at each formulation ratio, with the exception of OPO, whose content is slightly higher when it replaces the P-CFE extract (Table 2).

In both formulation ratios, the color of the BGs in which the HG* phase is loaded with the I-CFE extract appears dark brown, whereas the BG containing only the OG* phase loaded with the P-CFE extract shows a lighter brown tone. The dark brown is more intense in the 70:30 ratio (Figure 6a,c), where the I-CFE accounts for 68.60% of the BG composition (Table 2). Conversely, the light brown becomes even paler—almost cream-colored—in the 30:70 ratio (Figure 6b,d), where the P-CFE represents only 1.40% of the BG composition. This results in significantly higher lightness (L*) and yellowness (b*) values in BGs HG70:OG30* and HG30:OG70* (Table 7), both of which lack the I-CFE extract. In contrast, a significantly lower lightness is observed in BGs containing I-CFE and lacking P-CFE (HG70*:OG30 and HG30*:OG70, respectively). In any case, these BGs are developed as fat replacers for incorporation into healthier meat products, and the color provided by both extracts is highly suitable for this purpose.

In turn, Figure 6 provides a visual assessment of the self-standing ability (i.e., physical stability) of all BGs after 3 and 21 days of formulation. At day 3, BGs in both 70:30 and 30:70 HG:OG ratios (Figure 6a,b) showed no flow during the inverted vial test, clearly demonstrating their ability to support their own weight under gravity and confirming their classification as self-standing semi-solid structures. In both HG:OG ratios, this self-standing nature likely results from the three-dimensional network formed by the structuring agents—SA in the HG phase and MW in the OG phase—as previously reported for BGs developed with pectin-based HG and flaxseed–beeswax-based OG [26]. However, after 21 days of refrigeration, the HG70:OG30 BG (Figure 6c) exhibited structural disruption, failing to maintain a non-flowing texture, in spite of its higher enthalpy value (Table 6), which is reflecting greater molecules packing in the MW crystal assembly, and as also suggested by PLM images (discussed below). Therefore, this BG could no longer be considered a gel at this time point. This result is attributed to the absence of I-CFE extract in the HG phase of this formulation, which otherwise enhances stabilization and contributes significantly to the interconnection of the overall BG gel network throughout time when the HG phase predominates, also contributing to better homogeneity. This result is in consonance with the lowest density (lower *σ*_max_ value) obtained for the formed network in HG70:OG30 BG (Table 3). The absence of I-CFE appears to hinder over time the proper maintenance of OG droplets into a weaker HG polymeric network. Similar lack of self-standing ability has also been reported for other BGs with unbalanced phase ratios or insufficient gelator concentration [26,32]. In contrast, when the inverted vial test was performed three weeks after preparation, the remaining BGs showed good physical stability over time (Figure 6c,d).

The total loss (%) of each BG, which primarily consisted of OPO loss rather than water loss, is also included in Table 7. This result suggest that the instability mechanism is mainly associated with the release of the oil phase, rather than water migration. As expected, these losses were slightly higher in BGs formulated at the 30:70 ratio compared to those at the 70:30 ratio, which can be attributed to the higher OPO content in the OG phases of the 30:70 formulations (Table 2). In turn, at each ratio, the total loss was lower when the I-CFE extract was not present in the HG and was replaced by water (Table 7). The highest total loss was observed in BG HG30*:OG70* (8.25%), which contained 59.60% OPO and 29.40% I-CFE. These results suggest that, even in this latter case, the high proportions of oil and water present in the BG were effectively retained, with all samples exhibiting full stability under high centrifugal forces and over time (Figure 6c,d) with the mentioned exception. Total loss values recorded in this study (ranging from 0.00% to 8.25%) fall within the typical range reported for bigels formulated using a carrageenan/locust bean gum-based HG and a glyceryl monostearate-based OG [32], as well as those produced using gelatin and waxes, where solvent loss remained below 10% [9].

### 3.4. Polarized Light Microscopy of Bigels

**Figure 7:**
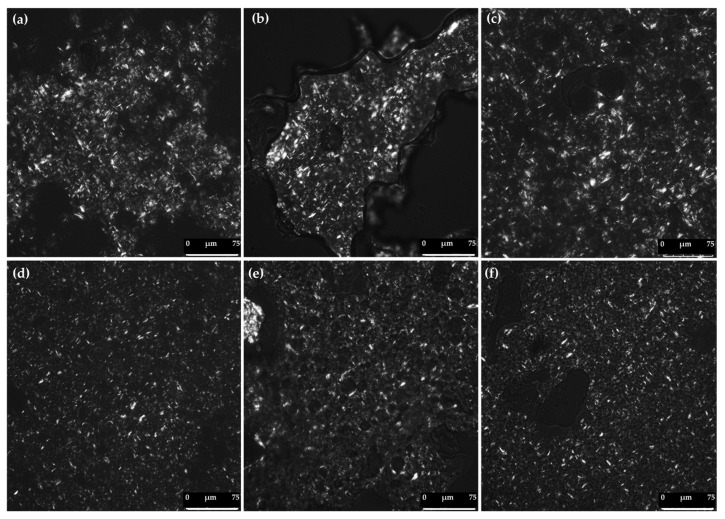
Polarized light microscopy of bigels (BGs): (**a**) HG70*:OG30 BG, at 70:30 ratio and loaded with I-CFE; (**b**) HG70:OG30* BG, at 70:30 ratio and loaded with P-CFE; (**c**) HG70*:OG30* BG, at 70:30 ratio and loaded with I-CFE and P-CFE; (**d**) HG30*:OG70 BG, at 30:70 ratio and loaded with I-CFE; (**e**) HG30:OG70* BG, at 30:70 ratio and loaded with P-CFE; (**f**) HG30*:OG70* BG, at 30:70 ratio and loaded with I-CFE and P-CFE. Scale corresponds to 75 μm.

The microstructural characteristics of the BGs were analyzed using polarized light microscopy (PLM) to evaluate the formation of the crystalline network influenced by HG:OG ratio and the presence or absence of CFEs (I-CFE and/or P-CFE) (Figure 7). According to the PLM images, the OG phase acted as the dispersed phase, while the HG served as the continuous phase in the 70:30 ratio (Figure 7a–c), and vice versa in OG-rich BGs (30% HG) (Figure 7d–f). Therefore, phase inversion occurred between these formulations at different HG:OG ratio. Similar observations have been reported for lycopene-loaded [6] and lutein-loaded bigels [7]. PLM images indicate that BGs exhibit both amorphous and crystalline characteristics derived from the individual HG and OG phases, respectively. Crystals in the OG phase appeared bright due to birefringence, whereas the HG phase appeared dark under PLM. All BGs showed needle-like crystalline structures, similar to those observed in other natural waxes used in the development of both OGs and BGs [27,33,34].

The results revealed that the main differences in crystal size and distribution were attributed to the varying HG:OG ratios rather than the presence or absence of CFEs. BGs with higher OG content exhibited smaller crystals compared to those rich in HG. The presence of smaller crystals promoted a more homogeneous and uniform distribution of the overall BG network, which was reflected in the improved rheological properties observed in the 30:70 ratio BGs (Figure 7d–f; Table 3, Table 4 and Table 5). Among the BGs with this ratio, the HG30*:OG70* sample appeared to exhibit a denser crystalline network, which could be attributed to the presence of both I-CFE and P-CFE, as previously discussed in the Section 3.1. Furthermore, in the 70:30 ratio, the incorporation of I-CFE into the HG also contributed to a more continuous and homogeneous distribution (Figure 7a, c) compared to the BG without this extract (Figure 7b), which, after three weeks of refrigerated storage, exhibited structural disruption and failed to maintain a non-flowing texture (Figure 6c).

## 4. Conclusions

This study confirms that the rheological and structural properties of BGs are strongly influenced by the HG:OG ratio and the presence of CFEs. All systems exhibited viscoelastic behavior, with the BGs at a 30:70 ratio showing greater mechanical strength and viscoelasticity, closely resembling the OG* phase. In contrast, the BGs at a 70:30 ratio had a creamier consistency, particularly when loaded with the I-CFE (inositol-rich) extract. The OG phase acted as an interacting filler, reinforcing the network regardless of extract type. The P-CFE (phenolic-compound-rich) extract notably enhanced elasticity in the BGs with higher HG content, while the combination of both extracts in a 30:70 ratio produced the strongest synergistic effect. These findings support the use of BGs as fat replacers in low-fat meat products, with 30:70 systems offering mechanical strength and 70:30 systems contributing to spreadability and flexibility. The strategic use of CFEs and tailored HG:OG ratios enables the design of structured systems with desirable functional attributes. Future studies will focus on BGs with intermediate ratios (e.g., 50:50) as well as on evaluating bioaccessibility, bioavailability, and sensory performance in real meat matrices.

## Figures and Tables

**Table 1:** Formulation of prepared bigels (BGs) and individual hydrogel (HG*) and oleogel (OG*) phases.

Bigel (BG)	HG:OG Ratio	HG	OG
HG70*:OG30	70:30	With I-CFE	Without P-CFE
HG70:OG30*	70:30	Without I-CFE	With P-CFE
HG70*:OG30*	70:30	With I-CFE	With P-CFE
HG30*:OG70	30:70	With I-CFE	Without P-CFE
HG30:OG70*	30:70	Without I-CFE	With P-CFE
HG30*:OG70*	30:70	With I-CFE	With P-CFE
**Control individual phases**			
HG*	100:0	With I-CFE	-
OG*	0:100	-	With P-CFE

An asterisk next to the name of a specific phase indicates that it was loaded with carob fruit extract: I-CFE (inositols-rich) for HG* and P-CFE (phenolic-rich) for OG*.

**Table 2:** Percentages of the different ingredients in bigels (BGs) that constitute the individual HG and OG phases, depending on the formulated HG:OG ratio.

	Individual HG Phase			Individual OG Phase			
Bigel (BG)	I-CFE	SA	Water	MW	Soy Lecithin	OPO	P-CFE
HG70*:OG30	68.60	1.40	0.00	3.75	0.075	26.18	0.00
HG70:OG30*	0.00	1.40	68.60	3.75	0.075	25.58	0.60
HG70*:OG30*	68.60	1.40	0.00	3.75	0.075	25.58	0.60
HG30*:OG70	29.40	0.60	0.00	8.75	0.175	61.08	0.00
HG30:OG70*	0.00	0.60	29.40	8.75	0.175	59.68	1.40
HG30*:OG70*	29.40	0.60	0.00	8.75	0.175	59.68	1.40

An asterisk next to the name of a specific phase indicates that it was loaded with carob fruit extract: I-CFE (inositols-rich) for HG* and P-CFE (phenolic-rich) for OG*.

**Table 3:** Critical (maximum) rheological properties at 25 °C defining the limits of the linear viscoelastic range (LVR) for developed bigels (BGs) and individual hydrogel (HG*) and oleogel (OG*) phases.

System	*σ*_max_ (Pa)	*γ*_max_ (%)	G*_max_ (kPa)	tan *δ* (-)
HG70*:OG30	2.28 ± 0.00 ^C^	0.10 ± 0.01 ^C^	1.91 ± 0.21 ^A^	0.44 ± 0.00 ^C^
HG70:OG30*	0.50 ± 0.00 ^A^	0.01 ± 0.00 ^A^	3.72 ± 0.83 ^B^	0.27 ± 0.03 ^A^
HG70*:OG30*	0.82 ± 0.00 ^B^	0.06 ± 0.01 ^B^	1.50 ± 0.30 ^A^	0.37 ± 0.03 ^B^
HG30*:OG70	31.64 ± 0.01 ^A^	0.03 ± 0.00 ^B^	107.34 ± 7.67 ^A^	0.20 ± 0.00 ^B^
HG30:OG70*	35.50 ± 0.00 ^B^	0.03 ± 0.01 ^B^	105.42 ± 16.39 ^A^	0.18 ± 0.01 ^A^
HG30*:OG70*	31.63 ± 0.00 ^A^	0.02 ± 0.00 ^A^	173.85 ± 11.25 ^B^	0.17 ± 0.00 ^A^
HG*	1.29 ± 0.00	2.80 ± 0.32	0.05 ± 0.01	0.39 ± 0.03
OG*	100.00 ± 0.00	0.03 ± 0.01	319.15 ± 83.15	0.20 ± 0.01

Mean value (*n* = 3) ± standard deviation. *σ*_max_, critical value of shear stress; *γ*_max_, critical value of shear strain; G*_max_, critical value of complex modulus (G* = [(*G*′)^2^ + (*G*″)^2^]^0.5^); tan *δ*, critical value of loss factor (tan *δ* = *G*″/*G*′). ^A–C^ For the same HG:OG ratio, different letters in the same column indicate significant differences (*p* < 0.05).

**Table 4:** Mechanical spectra data at 1 Hz and at 25 °C, and weak gel model parameters for developed bigels (BGs) and individual hydrogel (HG*) and oleogel (OG*) phases.

System	*G*′ (kPa)	*G*″ (kPa)	tan *δ* (-)	*A* (kPa s^1/*z*^)	*z* (-)	*R*^2^ (Equation (1))
HG70*:OG30	2.48 ± 0.12 ^B^	0.93 ± 0.04 ^C^	0.38 ± 0.00 ^C^	2.75 ± 0.12 ^B^	4.76 ± 0.06 ^A^	0.99
HG70:OG30*	2.95 ± 0.11 ^C^	0.78 ± 0.01 ^B^	0.27 ± 0.01 ^A^	3.11 ± 0.11 ^C^	6.17 ± 0.52 ^B^	0.99
HG70*:OG30*	1.93 ± 0.05 ^A^	0.56 ± 0.01 ^A^	0.29 ± 0.00 ^B^	2.05 ± 0.02 ^A^	5.80 ± 0.16 ^B^	0.99
HG30*:OG70	99.25 ± 4.00 ^A^	17.99 ± 0.41 ^B^	0.18 ± 0.00 ^B^	100.23 ± 4.04 ^A^	9.74 ± 0.15 ^A^	1.00
HG30:OG70*	100.69 ± 1.92 ^A^	16.82 ± 0.12 ^A^	0.17 ± 0.00 ^A^	100.72 ± 1.88 ^A^	10.10 ± 0.14 ^B^	0.99
HG30*:OG70*	159.87 ± 3.10 ^B^	26.33 ± 0.28 ^C^	0.17 ± 0.00 ^A^	160.08 ± 2.72 ^B^	10.14 ± 0.13 ^B^	0.99
HG*	0.05 ± 0.00	0.02 ± 0.00	0.38 ± 0.01	0.05 ± 0.00	4.70 ± 0.17	0.97
OG*	411.03 ± 8.00	74.81 ± 2.02	0.18 ± 0.00	413.93±7.50	9.39±0.10	1.00

Mean value (*n* = 3) ± standard deviation. *G*′, elastic or storage modulus; *G*″, viscous or loss modulus; loss factor (tan *δ* = *G*″/*G*′). ^A–C^ For the same HG:OG ratio, different letters in the same column indicate significant differences (*p* < 0.05).

**Table 5:** Viscosities (25 °C) at 0.1, 10, 50 and 100 s^−1^ (*η*_0.1_, *η*_10_, *η*_50_, and *η*_100_), and power law parameters (*K* and *n*) of Equation (2) for developed bigels (BGs) and individual hydrogel (HG*) and oleogel (OG*) phases.

System	*η*_0.1_ (Pa s)	*η*_10_ (Pa s)	*η*_50_ (Pa s)	*η*_100_ (Pa s)	*K* (Pa s*^n^*)	*n* (-)	*R*^2^ (Equation (2))
HG70*:OG30	48.97 ± 3.17 ^B^	3.20 ± 0.16 ^B^	2.09 ± 0.11 ^C^	1.80 ± 0.10 ^C^	13.36 ± 0.90 ^B^	0.44 ± 0.01 ^A^	0.98
HG70:OG30*	7.88 ± 0.87 ^A^	2.94 ± 0.03 ^B^	1.71 ± 0.03 ^B^	1.28 ± 0.03 ^B^	4.95 ± 0.32 ^A^	0.74 ± 0.02 ^C^	0.99
HG70*:OG30*	10.97 ± 0.91 ^A^	1.72 ± 0.12 ^A^	1.13 ± 0.07 ^A^	1.01 ± 0.06 ^A^	4.85 ± 0.42 ^A^	0.58 ± 0.01 ^B^	0.98
HG30*:OG70	147.43 ± 7.61 ^B^	2.86 ± 0.16 ^B^	1.39 ± 0.07 ^A,B^	1.13 ± 0.04 ^A^	22.27 ± 0.94 ^B^	0.20 ± 0.01 ^A^	1.00
HG30:OG70*	117.53 ± 1.96 ^A^	2.49 ± 0.11 ^A^	1.28 ± 0.07 ^A^	1.08 ± 0.07 ^A^	18.77 ± 0.65 ^A^	0.22 ± 0.01 ^A^	0.99
HG30*:OG70*	122.40 ± 3.36 ^A^	2.84 ± 0.17 ^A,B^	1.53 ± 0.05 ^B^	1.31 ± 0.01 ^B^	20.24 ± 0.78 ^A^	0.24 ± 0.02 ^B^	0.99
HG*	6.07 ± 0.27	1.17 ± 0.08	0.96 ± 0.08	0.90 ± 0.07	2.86 ± 0.08	0.63 ± 0.03	0.97
OG*	129.35 ± 3.65	2.20 ± 0.24	1.08 ± 0.04	0.93 ± 0.12	18.66 ± 1.55	0.18 ± 0.01	1.00

Mean value (*n* = 3) ± standard deviation. *K*, consistency index; *n*, flow behavior index. ^A–C^ For the same HG:OG ratio, different letters in the same column indicate significant differences (*p* < 0.05).

**Table 6:** Thermal properties of developed bigels (BGs), individual oleogel phase with P-CFE (OG*), microcrystalline wax (MW) and olive pomace oil (OPO).

Sample	Onset Temperature (T_O_, °C)	Peak Temperature (T_P_, °C)	Enthalpy (ΔH_M_, J/g)
HG70*:OG30	40.66 ± 0.61 ^A^	47.31 ± 1.21 ^A^	0.33 ± 0.05 ^A^
HG70:OG30*	40.85 ± 0.24 ^A^	49.08 ± 0.83 ^A^	1.31 ± 0.01 ^B^
HG70*:OG30*	41.01 ± 0.11 ^A^	47.51 ± 0.35 ^A^	0.41 ± 0.02 ^A^
HG30*:OG70	42.39 ± 2.07 ^A^	48.68 ± 0.86 ^A^	0.99 ± 0.05 ^A^
HG30:OG70*	40.84 ± 0.05 ^A^	48.33 ± 0.16 ^A^	0.98 ± 0.03 ^A^
HG30*:OG70*	40.57 ± 0.16 ^A^	48.46 ± 0.18 ^A^	1.15 ± 0.02 ^B^
OG*	41.24 ± 0.00	50.88 ± 0.32	1.02 ± 0.01
MW	33.34 ± 2.96	51.42 ± 0.06	70.99 ± 1.41
OPO	-	-	-

Mean value (*n* = 3) ± standard deviation. ^A,B^ For the same HG:OG ratio, different letters in the same column indicate significant differences (*p* < 0.05).

**Table 7:** Physicochemical parameters of developed bigels (BGs) and carob fruit extracts (CFEs).

Sample	L*	a*	b*	Total Loss (%)
HG70*:OG30	26.91 ± 0.68 ^A^	4.61 ± 0.93 ^A^	−2.15 ± 0.37 ^B^	2.06 ± 0.37 ^B^
HG70:OG30*	44.96 ± 0.30 ^C^	5.04 ± 0.13 ^A^	10.61 ± 0.14 ^C^	0.00 ± 0.00 ^A^
HG70*:OG30*	34.06 ± 0.28 ^B^	5.25 ± 0.14 ^B^	−10.66 ± 0.15 ^A^	4.82 ± 0.36 ^C^
HG30*:OG70	29.59 ± 0.81 ^A^	5.20 ± 0.46 ^B^	4.19 ± 0.43 ^B^	4.16 ±0.87 ^B^
HG30:OG70*	55.39 ± 0.35 ^C^	4.61 ± 0.16 ^A^	13.58 ± 0.50 ^C^	1.64 ± 0.38 ^A^
HG30*:OG70*	36.56 ± 0.28 ^B^	7.71 ± 0.13 ^C^	−6.71 ± 0.11 ^A^	8.25 ± 0.54 ^C^
I-CFE	26.31 ± 0.40	5.11 ± 0.28	−4.92 ± 0.11	-
P-CFE	63.13 ± 0.52	8.41 ± 0.17	16.01 ± 0.15	-

Mean value (*n* = 10) ± standard deviation. ^A–C^ For the same HG:OG ratio, different letters in the same column indicate significant differences (*p* < 0.05).

## Data Availability

The raw data supporting the conclusions of this article will be made available by the authors on request to ensure appropriate academic use and interpretation.

## References

[1] Zampouni K., Dimakopoulou-Papazoglou D., Katsanidis E. (2024). Food-Grade Bigel Systems: Formulation, Characterization, and Applications for Novel Food Product Development. Gels.

[2] Shakeel A., Lupi F.R., Gabriele D., Baldino N., De Cindio B. (2018). Bigels: A Unique Class of Materials for Drug Delivery Applications. Soft Mater..

[3] Martín-Illana A., Notario-Pérez F., Cazorla-Luna R., Ruiz-Caro R., Bonferoni M.C., Tamayo A., Veiga M.D. (2022). Bigels as Drug Delivery Systems: From their Components to their Applications. Drug Discov. Today.

[4] Xie D., Hu H., Huang Q., Lu X. (2023). Influence of Oleogel/Hydrogel Ratios and Emulsifiers on Structural and Digestion Properties of Food-Grade 3D Printed Bigels as Carriers for Quercetin and Catechin. Food Hydrocoll..

[5] Zheng H., Mao L., Cui M., Liu J., Gao Y. (2020). Development of Food-Grade Bigels Based on κ-Carrageenan Hydrogel and Monoglyceride Oleogels as Carriers for β-Carotene: Roles of Oleogel Fraction. Food Hydrocoll..

[6] Zhu Q., Gao J., Han L., Han K., Wei W., Wu T., Li J., Zhang M. (2021). Development and Characterization of Novel Bigels Based on Monoglyceride-Beeswax Oleogel and High Acyl Gellan Gum Hydrogel for Lycopene Delivery. Food Chem..

[7] Kaimal A.M., Singhal R.S. (2023). A Bigel Based Formulation Protects Lutein Better in the Gastric Environment with Controlled Release and Antioxidant Profile than other Gel Based Systems. Food Chem..

[8] Loza-Rodríguez N., Millán-Sánchez A., López O. (2023). Characteristics of a Lipid Hydrogel and Bigel as Matrices for Ascorbic Acid Stabilization. Gels.

[9] Saffold A.C., Acevedo N.C. (2022). The Effect of Mono-Diglycerides on the Mechanical Properties, Microstructure, and Physical Stability of an Edible Rice Bran Wax–Gelatin Biphasic Gel System. J. Am. Oil Chem. Soc..

[10] Ghiasi F., Golmakani M.-T. (2022). Fabrication and Characterization of a Novel Biphasic System Based on Starch and Ethylcellulose as an Alternative Fat Replacer in a Model Food System. Innov. Food Sci. Emerg. Technol..

[11] Zampouni K., Filippou A., Papadimitriou K., Katsanidis E. (2024). Evaluation of Bigel Systems as Potential Substitutes to Partially Replace Pork Backfat in Semi-Dry Sausages. Meat Sci..

[12] Baltuonytė G., Eisinaitė V., Kazernavičiūtė R., Vinauskienė R., Jasutienė I., Leskauskaitė D. (2022). Novel Formulation of Bigel-Based Vegetable Oil Spreads Enriched with Lingonberry Pomace. Foods.

[13] Álvarez M.D., Saiz A., Herranz B., Cofrades S. (2024). Olive Pomace Oil Structuring for the Development of Healthy Puff Pastry Laminating Fats: The Effect of Chilling Storage on the Quality of Baked Products. Foods.

[14] Ikram A., Khalid W., Wajeeha Zafar K.U., Ali A., Afzal M.F., Aziz A., Faiz ul Rasool I., Al-Farga A., Aqlan F., Koraqi H. (2023). Nutritional, biochemical, and clinical applications of carob: A review. Food Sci. Nutr..

[15] Macho-González A., López-Oliva M.E., Garcimartín A., Sánchez-Muniz F.J., Benedí J. (2020). Carob Fruit Extract-Enriched Meat Improves Pancreatic Beta-Cell Dysfunction, Hepatic Insulin Signaling and Lipogenesis in Late-Stage Type 2 Diabetes Mellitus Model. J. Nutr. Biochem..

[16] Azab A. (2022). D-Pinitol—Active Natural Product from Carob with Notable Insulin Regulation. Nutrients.

[17] Gabriele D., de Cindio B., D’Antona P. (2001). A Weak Gel Model for Foods. Rheol. Acta.

[18] Zhou M., Li B., Wu A., Hu Z., Zhou L., Hu H., Fu D. (2025). Preparation of a Two-Phase Gel System Based on Gelatin Hydrogel and Beeswax/Rice Bran Wax Oleogel and Eutectic Phase Behavior of Beeswax and Rice Bran Wax in Soybean Oil. LWT-Food Sci. Technol..

[19] Eisinaitė V., Jasutienė I., Vinauskienė R., Leskauskaitė D. (2023). Development of Bigel Based Dysphagia-Oriented Products, Structured with Collagen and Carnauba Wax: Characterisation and Rheological Behaviour. Int. J. Food. Sci. Technol..

[20] National Dysphagia Diet Task Force (2002). Dysphagia diet task, and AD association. National Dysphagia Diet: Standardization for Optimal Care.

[21] Álvarez M.D., Cofrades S., Espert M., Sanz T., Salvador A. (2021). Development of Chocolates with Improved Lipid Profile by Replacing Cocoa Butter with an Oleogel. Gels.

[22] Mancini F., Montanari L., Peressini D., Fantozzi P. (2002). Influence of Alginate Concentration and Molecular Weight on Functional Properties of Mayonnaise. LWT-Food Sci. Technol..

[23] Solo-de-Zaldívar B., Tovar C.A., Borderías A.J., Herranz B. (2015). Pasteurization and Chilled Storage of Restructured Fish Muscle Products Based on Glucomannan Gelation. Food Hydrocoll..

[24] Richterová V., Gjevik A., Vaculík O., Vejrosta J., Pekař M. (2025). Impact of Collagen on the Rheological and Transport Properties of Agarose Hydrogels. Gels.

[25] Lupi F.R., De Santo M.P., Ciuchi F., Baldino N., Gabriele D. (2017). A Rheological Modelling and Microscopic Analysis of Bigels. Rheol. Acta.

[26] Kavya M., Priyanka V., Jacob A.R., Nisha P. (2025). Investigating the Influence of Hydrogel and Oleogel Ratios on Physico Chemical Characteristics, Microstructure, Rheology, and Texture of a Food Grade Bigel. Biomacromolecules.

[27] Quilaqueo M., Iturra N., Contardo I., Millao S., Morales E., Rubilar M. (2022). Food-Grade Bigels with Potential to Replace Saturated and Trans Fats in Cookies. Gels.

[28] Russo N., Avallone P.R., Grizzuti N., Pasquino R. (2024). Stable O/W Emulsions by combining Pluronic L64 and Sodium Alginate. Colloids Surf. A: Physicochem. Eng. Asp..

[29] Li A., Gong T., Houa Y., Yang X., Guo Y. (2020). Alginate-Stabilized Thixotropic Emulsion Gels and their Applications in Fabrication of Low-Fat Mayonnaise Alternatives. Int. J. Biol. Macromol..

[30] Jiang Q., Chen K., Cai Z., Li Y., Zhang H. (2024). Phase Inversion Regulable Bigels Co-Stabilized by Chlorella Pyrenoidosa Protein and Beeswax: In-Vitro Digestion and Food 3D Printing. Int. J. Biol. Macromol..

[31] Dassanayake L.S.K., Kodali D.R., Ueno S., Sato K., Marangoni A., Garti N. (2011). Physical Properties of Organogels Made of Rice Bran Wax and Vegetable Oils. Edible Oleogels: Structure and Health Implications.

[32] Martins A.J., Guimarães A., Fuciños P., Sousa P., Venâncio A., Pastrana L.M., Cerqueira M.A. (2023). Food-Grade Bigels: Evaluation of Hydrogel: Oleogel Ratio and Gelator Concentration on their Physicochemical Properties. Food Hydrocoll..

[33] Loza-Rodríguez N., Millán-Sánchez A., López O. (2023). A Biocompatible Lipid-Based Bigel for Topical Applications. Eur. J. Pharm. Biopharm..

[34] Oyom W., Strange J., Nowlin K., Tukur P., Ferdaus M.J., Faraji H., Tahergorabi R. (2025). Development and Characterization of Bigel Systems as Carriers for Thyme Essential Oil Utilizing Hydrogel from Chicken Processing By-Products for Food Applications. Int. J. Biol. Macromol..

